# Why Are Women Predisposed to Intracranial Aneurysm?

**DOI:** 10.3389/fcvm.2022.815668

**Published:** 2022-02-10

**Authors:** Milène Fréneau, Céline Baron-Menguy, Anne-Clémence Vion, Gervaise Loirand

**Affiliations:** Université de Nantes, CHU Nantes, CNRS, INSERM, l'institut du thorax, Nantes, France

**Keywords:** intracranial aneurysm, cerebral artery, circle of Willis, sex difference, gender, endothelium, estrogens

## Abstract

Intracranial aneurysm (IA) is a frequent and generally asymptomatic cerebrovascular abnormality characterized as a localized dilation and wall thinning of intracranial arteries that preferentially arises at the arterial bifurcations of the circle of Willis. The devastating complication of IA is its rupture, which results in subarachnoid hemorrhage that can lead to severe disability and death. IA affects about 3% of the general population with an average age for detection of rupture around 50 years. IAs, whether ruptured or unruptured, are more common in women than in men by about 60% overall, and more especially after the menopause where the risk is double-compared to men. Although these data support a protective role of estrogen, differences in the location and number of IAs observed in women and men under the age of 50 suggest that other underlying mechanisms participate to the greater IA prevalence in women. The aim of this review is to provide a comprehensive overview of the current data from both clinical and basic research and a synthesis of the proposed mechanisms that may explain why women are more prone to develop IA.

## Introduction

Intracranial aneurysm (IA) is defined as a localized dilation of cerebral arteries which preferentially forms at arterial bifurcation of the circle of Willis. IAs are thought to result from an abnormal thickening of the artery wall at sites where hemodynamic stress is high (1). Unruptured IAs are generally silent but become symptomatic when they rupture, causing subarachnoid hemorrhage, with mortality rates of about 30–40% and severe neurological dysfunction and disability in a great part of subarachnoid hemorrhage survivors (2–4).

Cellular and molecular mechanisms leading to IA formation and rupture are not fully elucidated, but risk factors such as familial history of IA, high blood pressure, cigarette smoking, alcohol consumption, and female sex have been clearly identified. Indeed, in contrast to most neurocardiovascular diseases, the incidence of IA is higher in women than in men, whereas most of the risk factors that include cigarette smoking, hypertension, atherosclerosis, and alcohol consumption are all more common among men (5–13).

Women are found to suffer two times as often from unruptured IAs as men. Whereas, the overall prevalence of unruptured IAs in study population is reported about 3%−4%, it reaches 6% in women, with a woman-to-man prevalence ratio of 1.57 (14). This woman-to-man prevalence ratio changes with age, from 1.1 in populations with mean age of 50 years to 2.2 for populations over the age of 50 years (14). In addition, the follow-up of a cohort of patients diagnosed with either ruptured or unruptured IAs showed that female gender is an independent risk factors for the formation of new IAs (7).

Despite these clinical data, studies that are specifically designed to explain and understand the reasons for this female predisposition to IA remain few. Clinical analyses primarily addressed the role of sex hormones, and preclinical studies performed in rodent models of IA have mainly focused on the effects of ovariectomy and/or estrogen treatments, and *in vitro* on hormone actions in vascular cell models. The synthesis of published data supports a possible role of sex-specific hormonal mechanisms in the pathogenesis of IA. Nevertheless, the particular features of IA in women suggest that the greater predisposition of women to IA relies on complex and probably multiple mechanisms, including a role for hemodynamic forces.

## Gender Difference in IA Features

### IA Number and Localization

There is no statistical difference between men and women regarding the size and the laterality of unruptured IAs (15–17). However, together with a higher susceptibility to IA formation compared to men, women are more likely to develop multiple IAs (17–22). Also, women exhibit about two times the rate of bilateral IAs than men (16, 17). In addition, the number of IAs rises in women of increasing age (19). Both female sex and postmenopausal state are found as independent risk factors for the formation of multiple IAs (19).

In women, unruptured IA aspect has been shown to change with age: women of premenopausal age have a higher numbers of aneurysm lobes, whereas those in women of postmenopausal have larger size (23).

**Figure 1:**
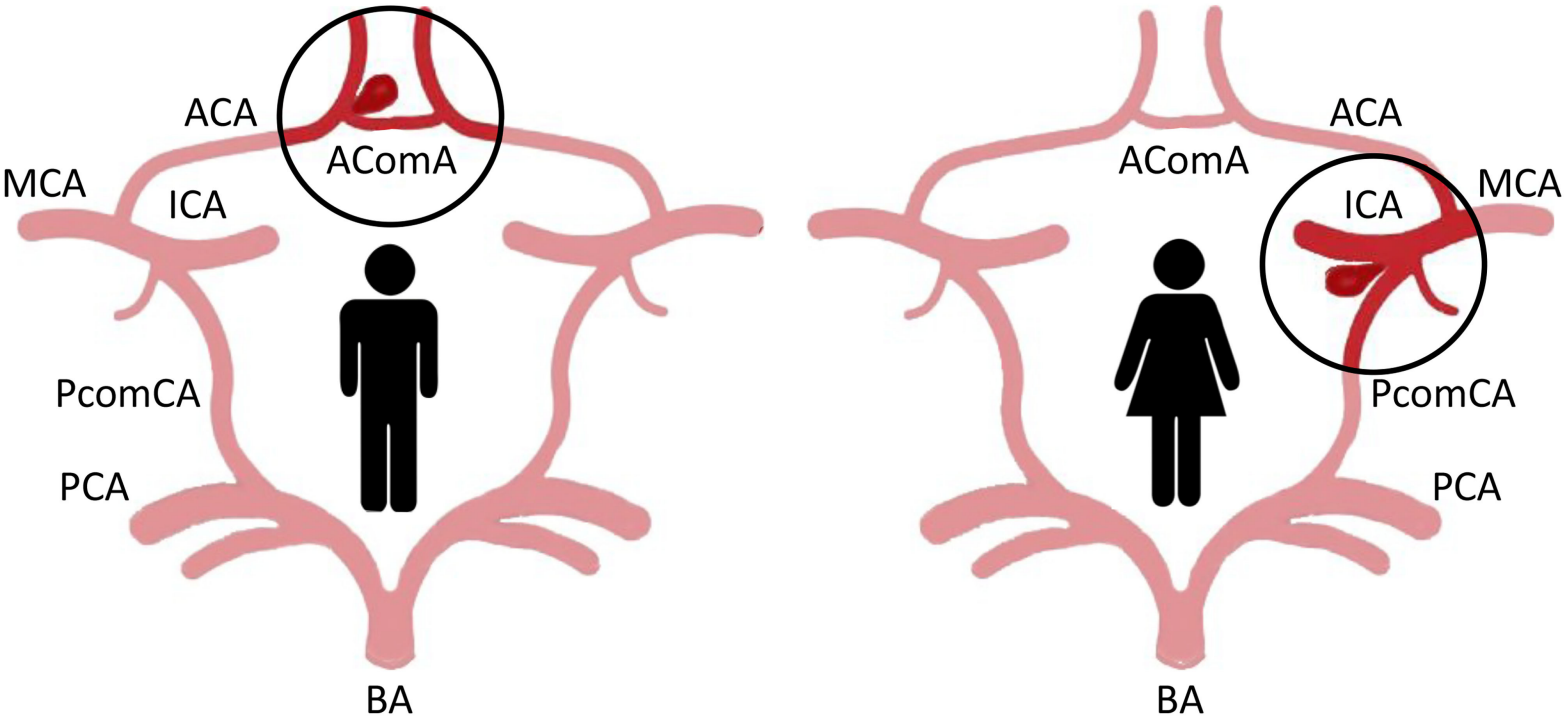
Major localization of IA in the circle of Willis of men and women. In men, IA affects more frequently the anterior cerebral artery (ACA) and anterior communicating artery (AComA; left). In women, IA localizes preferentially on the internal carotid artery (ICA), in particular at the bifurcation with the posterior communicating artery (PComA; right) (MCA, middle cerebral artery; BA, basilar artery; PCA, posterior cerebral artery).

A gender difference in the anatomical distribution of IA is also clearly demonstrated. In women, unruptured IA localizes preferentially on the internal carotid artery (ICA; 54% vs. 38% in men), whereas in men, IA affects more frequently the anterior cerebral artery (ACA; 29% vs. 15% in women) and anterior communicating artery (Figure 1) (15). No difference according to the gender has been observed in the frequency of IA in the middle cerebral artery and posterior circulation (posterior cerebral, basilar and vertebral, artery) (15).

### IA Growth and Rupture

As for IA formation, women are at increased risk for IA growth (7, 24). More particularly, female sex is shown to be an independent risk factor for the growth of unruptured IA in elderly patients (age ≥ 70 years) (25). However, the growth rate of an IA itself does not differ by sex (7).

Once an IA is formed, female sex does not represent a risk factor for its subsequent rupture (7). As a whole, no difference in the size of ruptured aneurysms between women and men has been detected (15, 20, 22, 26). However, some differences exist between ruptured IA in men and women. IA rupture and aneurysmal subarachnoid hemorrhage more frequently affect women than men but the gender distribution varied with age (15, 18, 27–29). Indeed, in young people, incidence of aneurysmal subarachnoid hemorrhage is slightly higher in men, and the increased risk of aneurysmal subarachnoid hemorrhage in women only appears after the fourth and fifth decades (28, 30–32). Accordingly, among patients with ruptured IAs, the mean age of women is higher (60–70 years) than that of men (50–60 years) (15, 16, 20, 26).

The location of aneurysmal subarachnoid hemorrhage also differs between women and men. According to the preferential location of IA on internal carotid artery in women, the posterior communicating artery is also the most common site of IA rupture in women, whereas anterior communicating artery aneurysm ruptures are overrepresented in men (16, 18, 20, 26, 33–35). This is in agreement with the majority of anterior communicating artery aneurysms in men and their higher risk of rupture than IAs at other locations (18, 36). Regarding this specific IA location, women exhibit a lower rate of ruptured anterior communicating artery aneurysms than men in whom these IAs are larger (16, 33). This greater IA size may thus participate to the higher proportion of men with ruptured anterior communicating artery.

For both women and men, outcomes varied according to the location of aneurysmal subarachnoid hemorrhage but the overall outcomes after IA rupture are similar in women and men (16, 20, 26, 33).

## Possible Causes of the Gender Difference in IA Formation and Rupture

### Anatomical and Hemodynamic Parameters

Both IA formation and rupture did not occur on same arteries in women and men. ICA and ACA have been identified as the main sites of IA formation and rupture in women and men, respectively. Indeed, there is a female preponderance of IA in all intracranial arteries except the ACA. It is well admitted that hemodynamic stress, such as high blood pressure or strong wall shear stress, may participate to IA formation and growth (37), which may suggest that gender difference in the arterial geometry and consequent arterial wall shear stress could participate in the different preferential location of IA in women and men.

**Figure 2:**
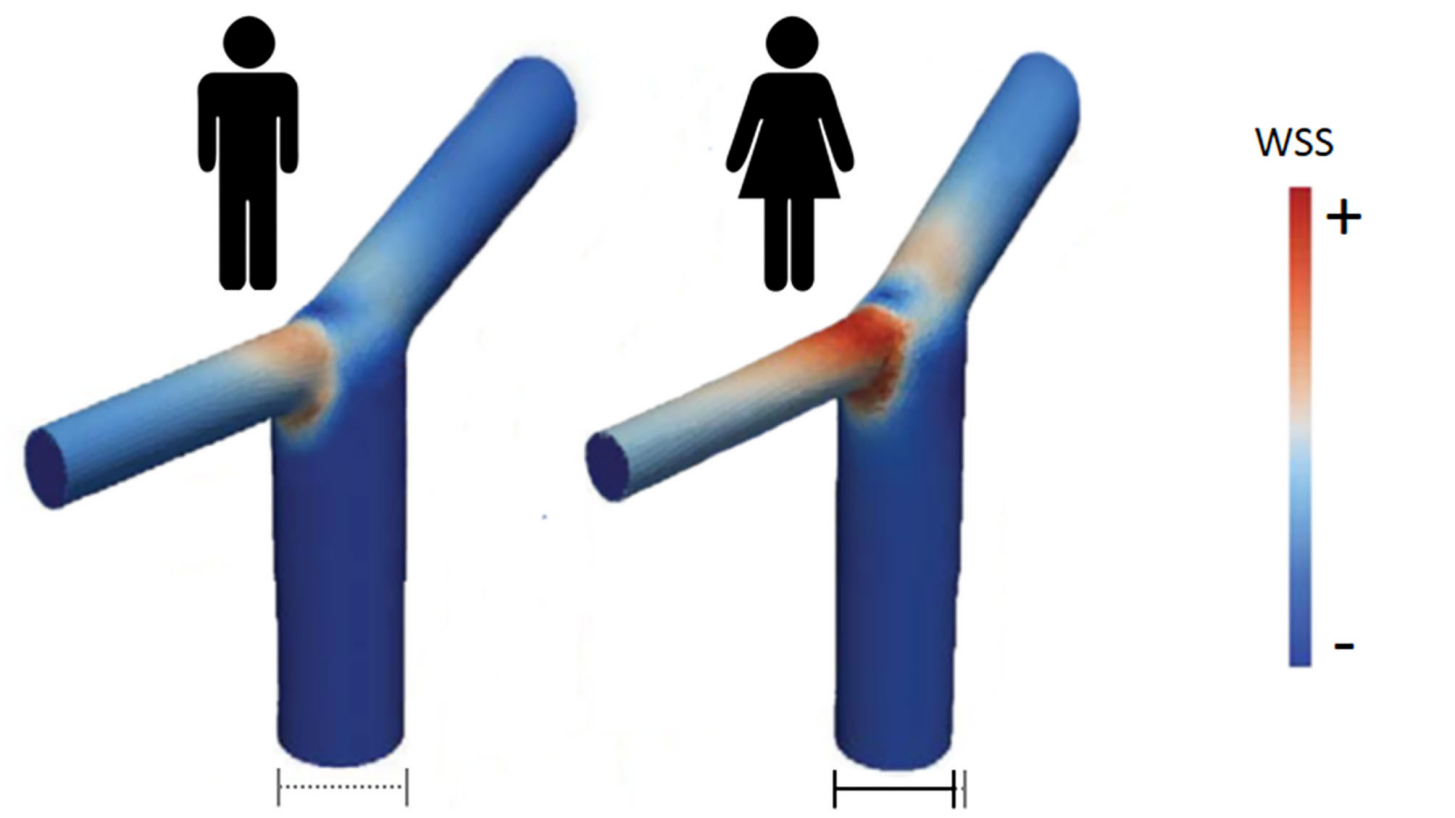
Wall shear stress intensity in ICA bifurcation in men (left) and women (right). According to computational fluid dynamics simulations, area of high WSS is larger and of stronger intensity in women than in men [adapted from Lindekleiv et al. (41)].

Analysis of the anatomical variations in the circle of Willis in more than one hundred of patients with IA by magnetic resonance angiography suggested a correlation between the sex-linked difference in IA distribution (preferential ICA aneurysm in women) and a sex-linked difference in anatomical variations of the circle of Willis (38). Beyond these anatomical variations, measurement of the diameter of arteries of the circle of Willis revealed that ICA, ACA, posterior cerebral artery and basilar artery were significantly smaller in women than in men, with the greatest difference found for ICA (39, 40). In contrast, the diameter of posterior communicating artery has been found to be either larger or similar in women compared to men. Since a smaller arterial diameter results in higher blood flow velocity and shear stress, arteries in women are expected to be submitted to stronger wall shear stress and tension than in men. Examination of the dimension and geometry of the terminal bifurcation of the ICA confirmed that the diameter of the parent artery and the branches is smaller in women than in men, but the bifurcation angle is the same in both sexes (41). Modeling of bifurcations and computational fluid dynamic simulations allowed to demonstrate that the maximum wall shear stress in the ICA bifurcation in the female was 50% higher than in men (41). In addition, the area of increased wall shear stress at the ICA bifurcation is larger in women compared to men (Figure 2). Such differences between men and women, although less pronounced, were also found at the bifurcation of the MCA into two main branches (41).

Regarding the wall tension generated by pressure, blood flow modeling of circle of Willis circulation has demonstrated that peak pressure is higher when artery diameter is smaller and the angle of the bifurcations is asymmetric (42).

All these observations thus support the idea that the gender difference in the diameter and geometry of bifurcations of arteries of the circle of Willis results in higher shear stress and peak pressure in women that may induce more severe endothelial damage and favors IA formation in women, particularly at ICA bifurcation and ICA posterior communicating artery junction. Moreover, it has been described that the larger the diameter of an IA relative to the native artery diameter, the higher the risk of rupture (43). With smaller diameter intracranial arteries in women, it can be thus considered that at equal IA size, the risk of rupture will be higher in women than in men.

### Hormones

Hormones are a fundamental part of sex differences and hormonal changes in the course of female life participate in sex differences in neurocardiovascular disease prevalence. Although the female preponderance of both unruptured and ruptured IAs in the general population is clear, and even more pronounced in familial forms of IA (44, 45), an important additional factor to be considered is the age. The change in the preponderance of IAs between men and women starts after the first two decades of life and became significant after the age of 55, with the peak in female prevalence of IA in the sixth decade (5, 14, 20, 28, 30–32). These changes are contemporary with the fall in estrogen levels occurring during and after menopause, which suggests the possible protective effect of estrogens on IA formation and rupture. This hypothesis is further supported by the greater risk of IA in association with earlier age at menopause (46). In contrast, women who used oral contraceptive pills and hormone replacement therapy are less likely to have cerebral aneurysms (47). The decline in estrogen concentration in peri- and post-menopause periods can thus be responsible of changes in cerebral artery structure and functions that favor the formation and/or the rupture of IAs.

Animal models of aneurysm provided a useful way to address the sex difference in IAs, in particular to understand the role of estrogens thanks to the use of ovariectomized females and/or estrogen supplementation. Estrogen effects are mediated by the activation of two nuclear estrogen receptors, ERα and ERβ, acting as transcription factors which control gene expression, and through a more recently described membrane G protein-coupled estrogen receptor (GPER) (48). Several studies have demonstrated the presence of functional ERα and ERβ in human and animal vascular smooth muscle and endothelial cells. However, vascular effects of estrogens are predominantly mediated by ERα (48).

In rodent models of IA, the incidence of IA is higher in female animals than in males and was further significantly increased in ovariectomized females, despite similar or even lower systolic blood pressure in females (6, 49–51). Surgically or pharmacologically induced-estrogen deficiency also aggravated IA lesions and significantly increased rupture of IAs (49, 52). In ovariectomized hypertensive rats, the increased incidence of carotid ligation-induced IA can be reversed by bazedoxifene, a selective estrogen receptor modulator, without change in blood pressure. This effect is associated with a restoration of ERα and ERβ expression in cerebral arteries that were downregulated by ovariectomy (53).

Estrogen treatment and specific estrogen ERβ agonist, but not ERα agonist, reversed the increased incidence of IA in ovariectomized female mice, which suggests that the protective effect of estrogens on IA was mediated by ERβ activation (50, 54). This role of ERβ was further confirmed by showing that the effect of the ERβ agonist was not observed in ERβ knockout mice and that non-ovariectomized ERβ knockout mice displayed an increased incidence of IA compared to non-ovariectomized control mice (50). With a protective effect of estrogens on IA mostly attributed to ERβ, the cerebral circulation stands out from the rest of the arteries in which the protective effect of estrogens is mediated by ERα.

## Molecular Mechanisms of Estrogen Protection to IA Formation

Although the exact pathogenesis of IA formation, growth, and rupture remains to be established, current knowledge suggests that endothelial dysfunction induced by hemodynamic injury at bifurcation of intracranial artery could be the initial step of IA formation (55–58). This first event then triggers a vascular inflammation process, with neutrophil and macrophage infiltration, oxidative stress, fragmentation of the internal elastic lamina, and degradation of the extracellular matrix by metalloproteinase, endothelial, and smooth muscle cell apoptosis (56, 59). All these interconnected processes lead to the structural degradation and remodeling of the arterial wall responsible for the weakening and fragility of the arterial wall. Protecting effects of estrogens can thus result from an inhibitory action on one or more components involved in IA formation.

### Estrogen Effect on Endothelial NO Synthase and Cerebral Artery Vasoreactivity

**Figure 3:**
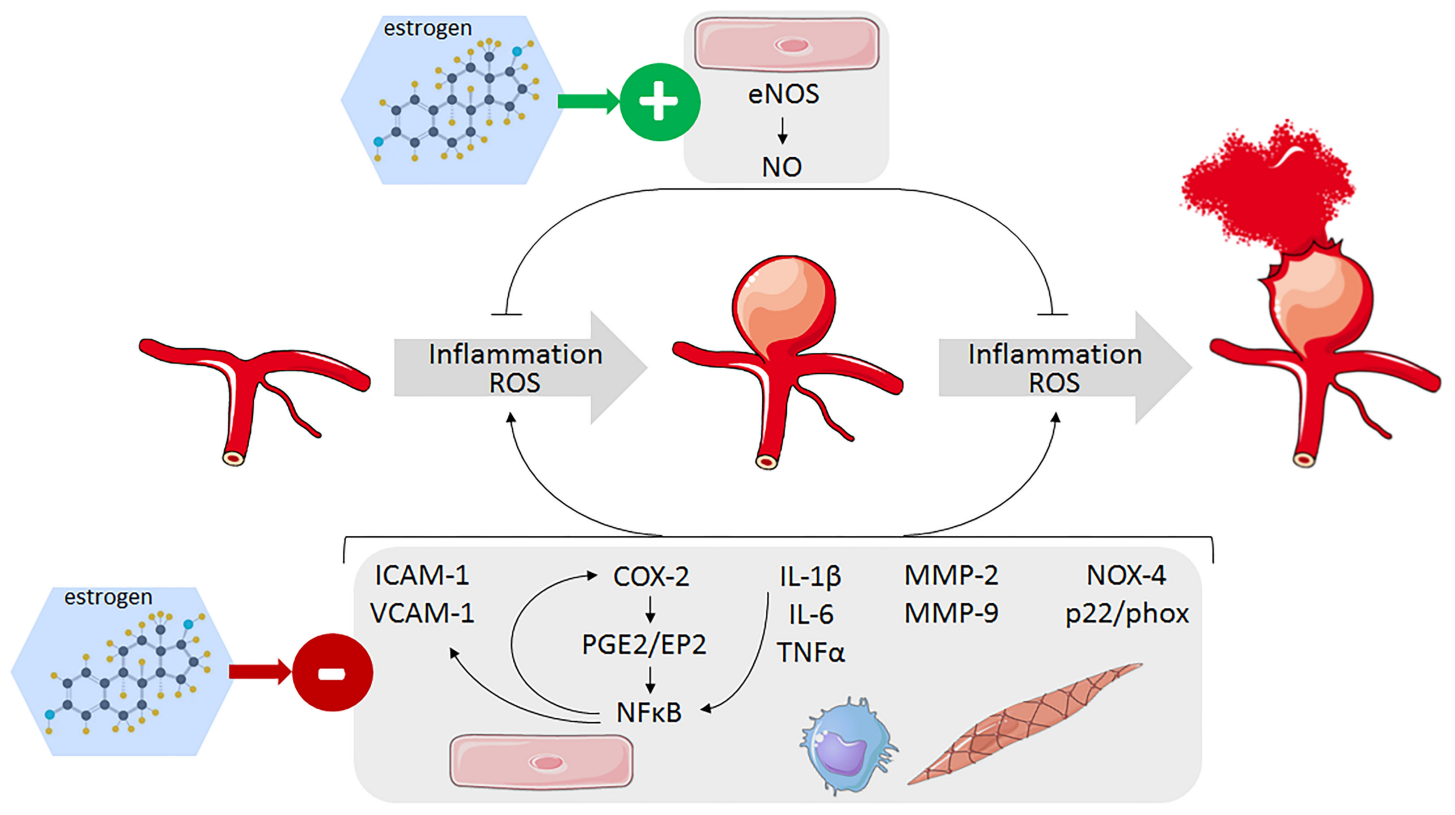
Identified pathways involved in the protective effect of estrogens against IA formation and rupture leading to decrease inflammation and oxidative stress in the arterial wall of cerebral arteries. Endothelial cells (pink), monocytes/macrophages (blue), and smooth muscle cells (light brow).

Endothelial dysfunction, with abnormal endothelial cell morphology and a loss of endothelial nitric oxide synthase (eNOS) expression, is a key early step of IA formation (Figure 3). In a rat model of IA, ovariectomy significantly decreased eNOS mRNA and protein expression, especially in the cerebral vascular wall of animals with saccular aneurysms (51). In contrast, estradiol treatment has been shown to increase the expression of eNOS in endothelial cells *in vitro* (51). Thus, estrogen deficiency promotes endothelial dysfunction whereas conversely, estrogen would protect against endothelial damage in the early phase of IA formation.

The *in vitro* effect of estrogen on eNOS expression in endothelial cell culture is mediated by ERα (51). ERα has been also shown to induce eNOS phosphorylation through phosphoinositide-3 (PI-3) kinase/Akt cascade leading to a rapid NO production in intact cerebral arteries from ovariectomized rats *ex vivo* and causing a long-term increase in NO production in the cerebral circulation of ovariectomized rats chronically treated with estrogen *in vivo* (60). This effect of ERα therefore seems to contradict the causal link established between the rise in NO production mediated by ERβ and the beneficial effect of estrogen on IA (50). Indeed, the protective effect of ERβ agonist on IA incidence in ovariectomized mice is completely abolished by the inhibition of eNOS by L-NAME treatment, which supports the fact that ERβ-induced NO production by eNOS mediates the beneficial effect of estrogens against IA formation (50). The *in vivo* role of ERα thus remains to be clarified but it has been shown that ovariectomy induces a loss of ERα expression in the vascular wall of mouse cerebral arteries with in contrast, an increase in ERβ expression (51), which may contribute to the discrepancy in the respective role of these two receptors in estrogen effects on NO production.

With regard to vasoreactivity, *ex vivo*, 17β-estradiol and agonists of ERα relax pressurized rat middle cerebral arteries from both male and female animals through a direct effect on smooth muscle cells (61). A relaxing effect of ERβ agonists was observed only in female rat arteries and was also due to an action on smooth muscle cells. ERβ agonists also induce relaxation of human cerebral artery in a NO-independent manner likely through an action on smooth muscle, whereas ERα receptor agonists have only a minimal effect (61).

In agreement with this relaxing effect of estrogens, ovariectomy enhanced the contractile response of rat cerebral arteries to vasoconstrictors, in association with an alteration of NO-dependent relaxing effect (62, 63). Tamoxifen or 17β-estradiol treatment, presumably through ERα, normalized cerebral artery reactivity to phenylephrine in ovariectomized rats (62, 63).

In summary, estrogens preserve normal endothelial function and have a limiting action on cerebral artery contraction and cerebrovascular tone through both endothelial-mediated NO dependent-relaxing effect and a direct relaxing action on smooth muscle, which participate to their protective effect on IA. Whereas, studies in ovariectomized rodent models of IA support a major role of ERβ, the observed changes in ERα and ERβ expression in cerebral artery wall of ovariectomized animals support a differential role of these receptors in the modulation of eNOS expression and activity, with a major of ERα before menopause and of ERβ after menopause.

### Estrogen Effect on Cerebral Artery Inflammation

The mechanisms linking high wall shear stress to the activation of proinflammatory signaling pathway at arterial bifurcation are not fully elucidated, but the transcription factor nuclear factor kappa B (NF-κB) is shown to play a critical role in IA formation and rupture (Figure 3). Its activation leads to an increase in the expression of vascular cell adhesion molecule-1 (VCAM-1), intercellular adhesion molecule-1 (ICAM-1), and monocyte chemoattractant protein-1 (MCP-1), which are responsible for the recruitment and adhesion of inflammatory cells to the endothelium where they produce proinflammatory cytokines such as tumor necrotizing factor alpha (TNFα), interleukin (IL)-1β, and IL-6 (56, 64–67). These cytokines then perpetuate local inflammation and neutrophil and macrophage infiltration in the cerebral artery wall, which produced damaging matrix metalloproteinases (MMP-2/9), and reactive oxygen species (ROS) (55). Whereas, the general vasculo-protector effects of estrogens are quite well documented, only a limited number of studies specifically addressed the anti-inflammatory action of estrogens on cerebral arteries and on IA.

Estrogens have been shown to limit proinflammatory cytokine expression and effects in cerebral arteries (Figure 3). Ovariectomy in female animals increased expression of TNFα and accumulation of neutrophils and macrophages in the arterial wall (49). Estrogen deficiency was also shown to upregulate IL-17A, which in turn downregulates E-cadherin and favors macrophage infiltration in the IA wall (52). Bazedoxifene decreases IL-1β mRNA expression in cerebral arteries which was upregulated by ovariectomy (53). Recently, a bioactive phytoestrogen daidzein, which reverses the increased IA incidence in ovariectomized mice via ERβ, was shown to decrease IL-6 mRNA level in cerebral arteries and, to a lesser extent, IL-1β and TNFα mRNAs (68). IL-6 level in the serum is increased and involved in the formation and rupture of IA in estrogen-deficient mice but not in control mice, which suggests that estrogen-induced repression of IL-6 expression participates to the beneficial effect of estrogen on IA (67).

Estrogen not only reduces IL-1β expression, but also suppresses exogenous IL-1β-mediated induction of cyclooxygenase 2 (COX-2)/prostaglandin E2 (PGE2) pathway in cerebral blood vessels of ovariectomized rats (69). IL-1β induces COX-2 expression through the activation of NF-κB, and the observed inhibitory effect of estrogen has been ascribed to an inhibition of NF-κB activity (70). This result is particularly interesting as it has been proposed that COX-2/PGE2/NF-κB pathway in cerebral artery endothelium is responsible for high wall shear stress-induced endothelial cell damage and may be the link between hemodynamic stress and IA formation (71). The inflammatory PGE2 formation is catalyzed from arachidonic acid by the sequential action of COX-2 and prostaglandin E synthase-1 (PGES-1). COX-2 and prostaglandin E receptor 2 (EP2) mRNA expression was induced *in vitro* in endothelial cell cultures exposed to shear stress. In a mouse model of IA induced by elevated hemodynamic stress, expression of COX2 and EP2 is increased in the endothelial cell layer at early stage of IA formation. Inhibition or knockout of COX-2 or EP2 resulted in decreased NF-κB expression and a reduction of incidence of IA formation (71, 72). The induction of COX-2/PGE2/EP2 signaling activates NF-κB, thus creating a self-amplified feedback loop that prevents the resolution of this initial process and contributes to generate the chronic inflammation in the cerebral arterial wall enabling for IA formation and progression. The observed inhibitory action of estrogen on NF-κB (69) might thus limit the shear stress-induced amplification loop of the COX-2/PGE2/NF-κB pathway and the perpetuation of inflammation in cerebral artery wall.

### Estrogen Effect on Cerebral Artery Oxidative Stress

Vascular oxidative stress and increased production of reactive oxygen species (ROS) are considered as the common mechanisms of vascular dysfunction and arterial disease, including IA (73) (Figure 3). Oxidative stress is mainly caused by an imbalance of ROS production by prooxidative enzymes (nicotinamide adenine dinucleotide phosphate (NADPH) oxidase, xanthine oxidase or the mitochondrial respiratory chain) and antioxidant mechanisms (superoxide dismutase, glutathione peroxidase, heme oxygenase, catalase, and so on.). The resulting rise in ROS concentration reduces bioactive endothelial NO and inhibits eNOs, which favors monocyte and macrophage recruitment creating a proinflammatory environment which leads to the activation of MMPs, phenotypic conversion of vascular smooth muscle cells and apoptosis, and finally a harmful arterial wall remodeling.

Excessive production of ROS has been demonstrated in aneurysmal walls in rodent models of IA, in association with an increased expression of heme oxygenase-1 and NADPH oxidase subunits (NOX4, p22phox, p47phox), mainly in macrophages and smooth muscle cells, whereas superoxide dismutase 1 was downregulated (51, 74). Free radical scavenger treatment or p47phox deletion markedly reduced IA formation and inhibited enlargement and medial degradation of IA (74). Estrogen deficiency in a rat model of IA increased the expression of NOX4 and p22phox in IA walls, and in contrast, 17β-estradiol inhibited NOX4 and p22phox expression in cerebral endothelial cell culture, suggesting that NADPH oxidase regulation by estrogen might participate to the gender difference in IA prevalence (51).

Additional indirect evidence supporting a role of ROS in the sex difference in IA has been provided by the differential effect of cigarette smoking on IA in men and women. Smoking is a well-known risk factor of IA formation and rupture, which mainly acts by inducing ROS accumulation (75). However, cigarette smoking has a more severe impact on IA, particularly on IA rupture, in smoking women than in men (76, 77). A recent study showed that relatively young women (between 30 and 60 years) with a positive smoking history have a four-fold increased risk for having an incidental unruptured IA (78). These results are consistent with an antiestrogenic effect of cigarette smoking (20), which should become even more apparent after the menopause when endogenous estrogen production is decreased and thus have a greater impact on the risk of postmenopausal IA.

### Estrogen Effect on Cerebral Artery Matrix and Elastic Mechanical Properties

Vascular remodeling is an important process in the pathogenesis of IA characterized by the degradation of the internal elastic lamina of aneurysmal walls and dynamic modification of extracellular matrix components such as elastin, collagens, and proteoglycan leading to weakening of the arterial wall (79, 80).

Arterial wall undergoes postmenopausal extracellular matrix changes similar to those occurring in the skin and bones, including a decrease in collagen and water content that leads to thinning and loss of elasticity offering a favorable ground to IA (81, 82). These changes particularly affect the media, which is the layer richest in collagen and elastin of the arterial wall. In contrast, thanks to the positive effect of estrogens on connective tissue and its turnover, hormone replacement has morphological effect on the carotid arteries in postmenopausal women, preserving the thickness of the arterial media layer (83).

In rats with IA, an imbalance between MMP-9 and MMP-2 and their inhibitors TIMP-1 and TIMP-2 is responsible for extracellular matrix degradation in the arterial walls leading to the progression and rupture of IA (84, 85)(Figure 3). In the same experimental model, the reduction in the incidence of IA rupture produced by treatment with the ER modulator bazedoxifene is associated with a significant decrease of MMP-9 expression that, on the contrary, was upregulated by ovariectomy (53). Estradiol administration has been also shown to inhibit the formation of lipid peroxidation products and restore middle cerebral arterial viscoelasticity and compliance in aged female rats (86, 87). In a rabbit model of IA induced by carotid ligation, estrogen deficiency, in combination with hypertension, increases vessel length and tortuosity in the circle of Willis, probably by lowering the tolerance of vascular tissue to hemodynamic stresses caused by carotid ligation, making it more vulnerable to flow-induced aneurysmal remodeling (88).

## Potential Role of 15-Hydroxyprostaglandin Dehydrogenase (15-PGDH)

The key role of COX-2/PGE2/NF-κB pathway in IA pathogenesis and its participation in the sex difference of the disease was further supported by the sex difference in the effect of aspirin on IA and the potential role of the PGE2 degrading enzyme 15-PGDH. Interestingly, frequent use of aspirin decreased the risk of IA rupture more significantly in men than in women (89, 90). This difference in aspirin effect was reproduced in male and female mice in an experimental model of IA (89). The beneficial effect of aspirin in mice is associated with a decreased expression of inflammatory molecules in cerebral arteries, which has been ascribed to its inhibitory action of COX-2 (89). In an attempt to identify the mechanisms involved in the differential effect of aspirin on IA in male and female, gene expression analysis in cerebral arteries has revealed a lower expression of 15-PGDH and higher levels of proinflammatory molecules (COX-2, CD-68, MMP-9, MCP-1, and NF-κB) in treated females than in treated males. 15-PGDH is the main enzyme of prostaglandin degradation that stops the biological activity of PGE2 by converting it to 15-keto-PGE2, an endogenous peroxisome proliferator-activated receptor γ (PPARγ) agonist. Thus, even if the activity of COX-2 is reduced by aspirin, the low level of 15-PGDH in female could contribute to maintaining, at least in part the activity of the PGE2/NF-κB pathway. Indeed, expression of COX-2, CD-68, MMP-9, MCP-1, and NF-κB was higher in cerebral arteries of aspirin-treated female mice than in treated males, and this difference and also the increased risk of IA rupture between males and females were completely equalized by treatment a 15-PGDH activator (89). This observation further supports the essential role of 15-PGDH-mediating PGE2 degradation in the protective effect of aspirin. It also suggests that the low expression of 15-PGDH in cerebral artery in female might favor high shear stress-induced COX-2/PGE2/NF-κB pathway activation and the resulting maintained inflammation in arterial wall, thus participating to the increased propensity to IA rupture. Moreover, the low catalytic activity of 15-PGDH also limits the activation of PPARγ shown to decrease IA formation and rupture (91, 92).

## Conclusion

Experimental works driven over the past decades to understand the well-known higher prevalence of IA women compared with men have gathered knowledge that allows us to propose different mechanisms that would be involved. One of them lies in the anatomic difference of the circle of Willis between men and women, with diameters and geometry of bifurcation of the arteries leading to higher hemodynamic stresses in women, driving more severe endothelial damage which favors IA formation. In addition, cigarette smoking appears to have a greater impact on IA in women than in men, an effect that could be related to the low level of 15-PGDH described in cerebral artery in female, and as a consequence, a stronger prooxidative damaging action of smoking in women. However, although these unmodifiable and modifiable risk factors predispose women to IA, they are counteracted by the protective effects of estrogens, acting on multiple steps of IA formation including, endothelial dysfunction, inflammation, and oxidative stress. The loss of these estrogen-mediated protecting mechanisms at menopause thus plays a major role in the critical increase in IA prevalence in women over the age of 50 years.

It is obvious that multiple combined mechanisms are responsible for the gender differences of IA disease. As in any disease, sex differences in IA may be linked to sex hormones but also to nonhormonal factors dependent of the genes present in the X and Y chromosomes. Currently, the vast majority of studies, in humans and animal models, have focused on the role of sex hormones, more particularly of estrogens, and further research is needed to determine the part of each of these mechanisms in the female susceptibility to IA. Moreover, beside biological sex influences on IA pathophysiology, the psycho-sociocultural construct of gender can further participate to the difference between men and women. Societal, cultural, behavioral, and psychological factors may add to or modulate the biological factors involved in men and women differences toward IA formation and rupture.

Regarding IA patient care, despite the significant data that demonstrate the negative impact of female sex on IA incidence and rupture, this important variable is surprisingly largely neglected in clinical practice. However, even if the mechanisms involved are not elucidated, the current data would nevertheless make it possible to propose ways to improve the management of women suspected or diagnosed with IA. First, it would be relevant to consider women as a high-risk group. Second, given the strong impact of hemodynamic and oxidative stress on IA in women, the implementation of intensive strategies to lower blood pressure and promote cigarette smoking cessation seems to be strongly warranted in women.

There is no doubt that our understanding of the mechanisms underlying sex differences in IA will improve further in the coming years and contribute to a better understanding of the pathophysiology of IA. The challenge will then be to transform this knowledge into means to improve the prevention of IA formation, progression, and rupture, and more globally for a better care of IA patients, both women and men.

## Author Contributions

GL participated in the preparation of conceptualization, literature search, and writing—original draft preparation. A-CV and GL provided funding acquisition. MF, CB-M, A-CV, and GL involved in writing, designing, and making of the figures—draft revision. All authors have read and agreed to the published version of the manuscript.

## Funding

This work benefited funding from Fondation pour la Recherche Médicale, (DPC20171138968), Fondation Recherche Cardio-Vasculaire - Institut de France (Coeur de Femmes), and Programme d'Investissements d'Avenir ANR-16-IDEX-0007 (NExT Junior Talent, ICARA project, co-funded by Région Pays de la Loire and Nantes Métropole).

## Conflict of Interest

The authors declare that the research was conducted in the absence of any commercial or financial relationships that could be construed as a potential conflict of interest.

## Publisher's Note

All claims expressed in this article are solely those of the authors and do not necessarily represent those of their affiliated organizations, or those of the publisher, the editors and the reviewers. Any product that may be evaluated in this article, or claim that may be made by its manufacturer, is not guaranteed or endorsed by the publisher.
